# Visit-to-visit blood pressure variability and risk of chronic kidney disease: A systematic review and meta-analyses

**DOI:** 10.1371/journal.pone.0233233

**Published:** 2020-05-29

**Authors:** Huihui Li, Jing Xue, Wenjie Dai, Yusa Chen, Qiaoling Zhou, Wenhang Chen

**Affiliations:** 1 Department of Nephrology, Xiangya Hospital, Central South University, Changsha, Hunan, China; 2 Department of Scientific Research, Xiangya Hospital, Central South University, Changsha, Hunan, China; 3 Xiangya School of Public Health, Central South University, Changsha, Hunan, China; 4 Laboratory of Kidney Disease, Department of Nephrology, Hunan Provincial People’s Hospital, Hunan Normal University, Changsha, Hunan, China; University of Oxford, UNITED KINGDOM

## Abstract

**Objective:**

Previous studies have shown that visit-to-visit blood pressure variability (BPV) is associated with chronic kidney disease (CKD). However, the results have not been consistent among studies. This systematic review and meta-analysis was conducted to comprehensively assess the association between visit-to-visit BPV and the risk of CKD.

**Methods:**

Medline, Embase, and the Cochrane Library were searched from the date of inception through 1 August 2019 using the terms “blood pressure variability,” “chronic kidney disease,” “nephropathy,” and other comparable terms. The primary outcome was the development of CKD. Two reviewers extracted the data independently. Meta-analysis was performed using a random effects model.

**Results:**

Fourteen studies were included in the systematic review and meta-analysis. The risk of CKD was significantly greater in patients with high baseline systolic blood pressure variability (SBPV) than in patients with low baseline SBPV: the standard deviation (SD) showed relative risk (RR) of 1.69 and 95% CI of 1.38–2.08, the coefficient of variation (CV) showed RR of 1.23 and 95% CI of 1.12–1.36, and variance independent of mean (VIM) showed RR of 1.40 and 95% CI of 1.15–1.71. RRs for each unit increase in visit-to-visit SBPV and risk of CKD were 1.05 (95% CI: 1.03–1.07) for SD, 1.06 (95% CI: 1.03–1.09) for CV, and 1.1 (95% CI: 0.96–1.25) for VIM. Diastolic BPV was similarly predictive of CKD based on SD and CV.

**Conclusions:**

Increased visit-to-visit BPV might be an independent risk factor for CKD. However, significant heterogeneity was present; thus, future prospective studies are needed to confirm our findings. Our results indicate that treatment of hypertension should control blood pressure levels and prevent abnormal fluctuations in blood pressure to reduce the risk of CKD.

## Introduction

Chronic kidney disease (CKD) is a global public health problem that leads to poor health and high costs [1]. A cross-sectional survey of a national sample of Chinese adults showed that the overall estimated prevalence of CKD was 10.8% [2]. Therefore, a better understanding of the risk factors for impaired renal function would have a major impact on both clinical practice and public health.

High blood pressure (BP) has been identified as the leading risk factor for mortality worldwide and one of the most important risk factors for CKD [3, 4]. BP fluctuations may occur under the influence of various factors such as age, increased vascular stiffness, sympathetic nervous system activity, and nonadherence to treatment [5]. The link between visit-to-visit blood pressure variability (BPV) and morbidity or mortality events has been emphasized in recent years. A growing body of evidence has shown associations of BPV with stroke, cardiovascular disease, and all-cause mortality in high-risk populations [6–8]. In terms of renal outcomes, BPV is a strong risk factor for poor outcomes in patients undergoing hemodialysis [9]. Previous studies have found that increased BPV is a prognostic factor for the development, progression, and severity of renal outcomes [10–13]. Other studies have shown associations of BPV with renal outcomes, but have yielded inconsistent results [14, 15].

It is important to reduce the incidence of CKD by early identification of high-risk patients and modification of treatment-related risk factors. The impact of increases in BPV (i.e., above mean BP values) on the risk of CKD remains unclear. An improved understanding of this relationship might provide a potential novel target for prevention of CKD. Thus, this systematic review and meta-analysis of cohort studies was conducted to comprehensively assess the association between visit-to-visit BPV and the risk of CKD.

## Materials and methods

### Search strategy

This systematic review and meta-analysis was conducted in accordance with the Meta-analysis of Observational Studies in Epidemiology (MOOSE) guidelines [16]. Comprehensive searches were conducted in Medline, Embase, and the Cochrane Library (including the Cochrane Central Register of Controlled Trials) from the date of inception to 31 December 2019. The methodology complied with the Cochrane Handbook for Interventional Systematic Reviews [17]. The article was written in a manner that adhered to the Preferred Reporting Items for Systematic Reviews and Meta-Analyses (PRISMA) statement [18]. The following search terms were used: “blood pressure variability,” “chronic kidney disease,” “nephropathy,” and other comparable terms. Additionally, we manually searched the reference lists of the retrieved articles to identify additional possible clinical studies. The protocol for the meta-analysis was registered with PROSPERO (Website: https://www.crd.york.ac.uk/PROSPERO; Registration number: CRD42020149248).

### Study eligibility

Studies were included if the following criteria were fulfilled: (1) cohort design, including prospective cohort or retrospective cohort, or follow up study of randomized controlled trial; (2) the exposure of interest was BPV; (3) the outcome was CKD (including proteinuria, nephropathy, diabetic kidney disease, and end-stage renal disease); (4) quantitative estimates were reported for the adjusted relative risk (RR) and 95% confidence interval (95% CI) for CKD associated with BPV.

A study was excluded if the following criteria were met: (1) inclusion or exclusion criteria of the study were unclear or unreasonable; (2) insufficient data of interest were reported; (3) the study was a review, supplement, abstract only, commentary article, editorial, or grey literature. If relevant data from ≥2 articles were derived from the same cohort, only the study with a longer follow-up duration or larger population was included in this analysis. Discrepancies were resolved by discussion.

### Extraction of data

Data from all included studies were extracted by two independent review authors (H.L. and J.X.). Discrepancies in data abstraction were resolved through discussion or by a third investigator (W.C.). Data extracted from studies included the following information: first author’s name, publication year, country, database, study design, duration of follow-up, sample size, mean patient age, sex, outcomes, outcome definitions, BPV measurements (e.g., standard deviation [SD]; coefficient of variation [CV]; variability independent of the mean [VIM] of systolic BP, diastolic BP, or both), fully adjusted RRs, and 95% CIs.

### Assessment of study quality

The quality in prognosis studies (QUIPS) tool is recommended for quality assessment in reviews of prognostic factors [19]. Two reviewers (H.L. and J.X.) assessed the risk of bias in individual studies independently using the QUIPS tool [20]. This tool assesses six domains for bias and applicability of the research question: study participation, study attrition, prognostic factor measurement, outcome measurement, study confounding, and statistical analysis and reporting. An overall rating for each domain is rated as “high”, “moderate”, or “low” risk of bias. Discrepancies were resolved by discussion.

### Data synthesis and statistical analysis

All statistical analyses were conducted using Stata version 12 (Stata Corp., College Station, TX, USA). Risk estimates for each study were reported as odds ratio, risk ratio, or hazard ratio; these were presumed to be approximately equivalent for effect sizes <2.5 and follow-up <20 years and therefore were merged directly [6, 21, 22]. We used RR to measure the effect of the association between BPV and CKD. Risk estimates were pooled using a random effects model due to the observational design of the included studies.

To quantify the dose–response relationship between BPV and risk of renal disease, we calculated the RR for each unit increment in BPV in each study. For studies that reported risk estimates using ranges of BPV, we calculated the midpoint in each category by calculating the average of the lower and upper bounds. When the highest or lowest category was open-ended, we assumed that the open-ended interval was similar in length to the adjacent interval [23]. The generalized least-squares (GLST) method was used to estimate RRs [24, 25]. We treated BPV as a continuous variable and adopted linear models to calculate RRs for each 1 mmHg increase in BPV in each study. When RRs indicated specific intervals of BPV increments (e.g., 3 mmHg or 5 mmHg), RRs for a change of 1 mmHg were calculated with the corresponding root of the original value. For instance, if the reported RR for 5 mmHg increments was 1.20, the RR for 1 mmHg change would be the fifth root of 1.2, which is 1.04 [7, 26].

Heterogeneity among the included studies was evaluated using the Cochrane Q test and I^2^ statistic [27]. A value of I^2^ >50% was considered indicative of a significant level of heterogeneity; stratified syntheses and sensitivity analyses were used to explore sources of heterogeneity. Stratified analyses were performed based on follow-up duration, country, population, or sample size. Publication bias was assessed using Egger’s test [28]. A two-tailed P-value <0.05 was considered statistically significant.

## Results

### Literature search and characteristics of included studies

**Fig 1:**
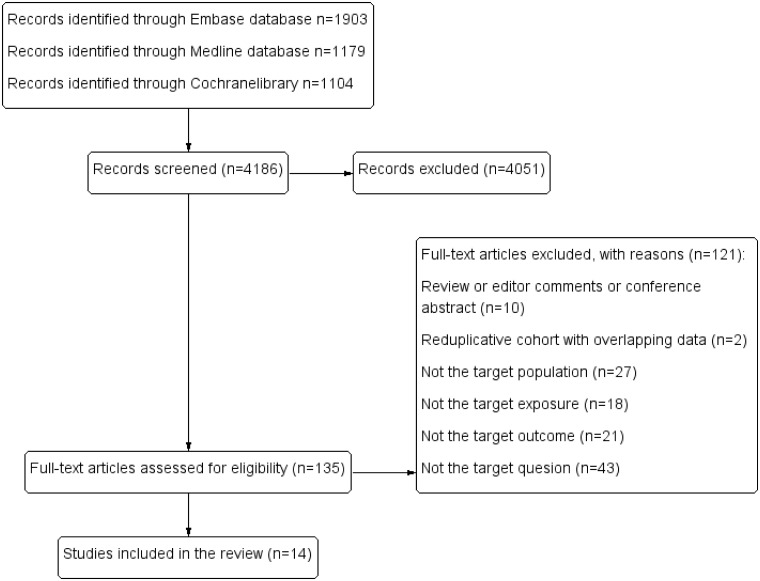
Flowchart of literature search.

The results of the literature search are summarized in Fig 1. Of 4186 references retrieved in our literature search, the titles and abstracts of 4051 publications indicated that they were clearly irrelevant; these irrelevant references were excluded. Thus, we obtained 135 full-text articles and fully reviewed them. Of these 135 articles, two described the same cohort study and presented overlapping data [29, 30]. Ultimately, 14 studies, with a total of 11,407,535 participants, fulfilled our inclusion criteria and were included in the research [10, 11, 13, 14, 31–40].

The characteristics of all selected studies are listed in Table 1. Of the 14 studies, the mean follow-up duration ranged from 2.6 to 11.5 years, and the mean patient age ranged from 28 to 68 years. Seven of these studies were conducted in Asia (three in Japan, two in China, and one each in Korea and Iran); the remaining seven were conducted in North America and Europe. One of the studies enrolled participants in 20 countries from Asia, Australia, Europe, and North America [36]. The populations of the studies included the general population, patients with hypertension, patients with type two diabetes, and patients with type one diabetes. All studies reported adjusted estimates. S1 Table in S1 Data showed the results of quality assessments using the QUIPS tool. Most of the included studies were deemed to be low or moderate risk of bias. Two of the studies was judged to be moderate risk of bias [10, 11].

**Table 1:** Characteristics of included cohorts.

Reference	Publication year	Country	Database	Study design	Population	No of subjects	Duration of follow-up (year)	Mean age (year)	Males (%)	Outcome	Definition
Yu and colleagues [31]	2019	China	Wonders Big data management and control platform	Retrospective	Type 2 diabetes mellitus patients	12630	3.9	61.4	41.5	Diabetic kidney disease	International Classification of Disease, tenth version (ICD-10) codes or a diagnosis of ‘diabetic kidney disease’ in the medical records
Viazzi and colleagues [32]	2019	Italy	The Italian Association of Clinical Diabetologists initiative	Retrospective	Type 2 diabetes mellitus patients	30851	4	65	57	Chronic kidney disease	eGFR less than 60 ml/ min per 1.73 m2, a reduction at least 30% from baseline and a combination of either one of the above endpoints
Li and colleagues [33]	2019	China	The Renal Sub-study of the China Stroke Primary Prevention Trial	Retrospective	Hypertensive patients	10051	4.4	59.5	36.5	Chronic kidney disease	A decrease in eGFR ≧30% and to a level of <60 mL/min/1.73 m2, or ESRD
										Rapid renal function decline	An average decline in eGFR of ≧5 mL/min/1.73 m2 per year
Bae and colleagues [34]	2019	Korea	The Korean National Health Insurance Service	Retrospective	General population	8199089	7.89	48.3	58.7	End-stage renal disease	ICD-10 codes, initiation of renal replacement therapy or kidney transplantation
Sohn and colleagues [35]	2016	USA	The US Department of Veterans Affairs healthcare system	Retrospective	Diabetic patients	208338	3.5	53.7	95.4	Nephropathy	Identified using ICD-9-CM codes
Ohkuma and colleagues [36]	2017	20 countries	The ADVANCE-ON study	Retrospective	Type 2 diabetes mellitus patients	9114	7.6	68	58	Major renal events	Chronic renal-replacement therapy and death from renal disease
Ceriello and colleagues [14]	2017	Italy	The Italian Association of Clinical Diabetologists initiative	Retrospective	Type 2 diabetes mellitus patients	11791	2.6–3.4	NP	56.3	Albuminuria	Development of albuminuria in patients with normoalbuminuria at baseline
										GFR below 60 mL/min/1.73 m2	Decrease in GFR below 60 mL/min/1.73 m2 in patients with GFR ≥ 60 mL/min/1.73 m2 at baseline
Whittle and colleagues [37]	2016	USA	The Antihypertensive and Lipid-Lowering Treatment to Prevent Heart Attack Trial	Retrospective	Hypertensive patients	21245	3.5	66.6	54.7	End-stage renal disease	Identified using the US Renal Data System
										≥50% decline in eGFR	
Gosmanova and colleagues [38]	2016	USA	The cohort of U.S. veterans	Retrospective	U.S. veterans	2865157	4.9	60	94	End-stage renal disease	Initiation of renal replacement therapy
Yano and colleagues [39]	2015	Japan	The Specific Health Check and Guidance System	Prospective	General population	48587	3	61.7	39	Chronic kidney disease	The presence of proteinuria or eGFR<60 mL/min per 1.73 m2
Takao and colleagues [40]	2014	Japan	Hospital-based cohort	Retrospective	Type 2 diabetes mellitus patients	664	11.5	-	-	Nephropathy	Development of microalbuminuria, i.e., UAE ≥30 mg/g Cr
										Progression of nephropathy	Decrease of eGFR to <45 ml/min/1.73 m2
Noshad and colleagues [11]	2014	Iran	Hospital-based cohort	Retrospective	Type 2 diabetes mellitus patients	194	2.6	51.7	52.1	Microalbuminuria	UAE between 30 and 299 mg per 24 h in at least two consecutive urinary protein measurements
Okada and colleagues [10]	2013	Japan	Hospital-based cohort	Retrospective	Type 2 diabetes mellitus patients	354	3.76	65.5	61.6	Albuminuria	UAE >30 mg per 24 h
Kilpatrick and colleagues [13]	2010	United Kingdom	The Diabetes Control and Complications Trial	Retrospective	Type 1 diabetes mellitus patients	1261	9	28	60.3	Nephropathy	The development of an AER≥40 mg/24 h (28 μg/ min) on any annual evaluation, providing that the baseline AER was <40 mg/24 h (28μg/min)

### Association between visit-to-visit BPV and risk of CKD

Visit-to-visit systolic BPV (SBPV) was defined using SD, CV, and VIM. The risk of CKD was significantly greater in patients with high baseline SBPV than in patients with low baseline SBPV (SD [RR = 1.69, 95% CI: 1.38–2.08; I^2^ = 98.8%; S1 Fig in S1 Data], CV [RR = 1.23, 95% CI: 1.12–1.36; I^2^ = 91.1%; S2 Fig in S1 Data], and VIM [RR = 1.40, 95% CI: 1.15–1.71; I^2^ = 95.9%; S3 Fig in S1 Data]).

**Fig 2:**
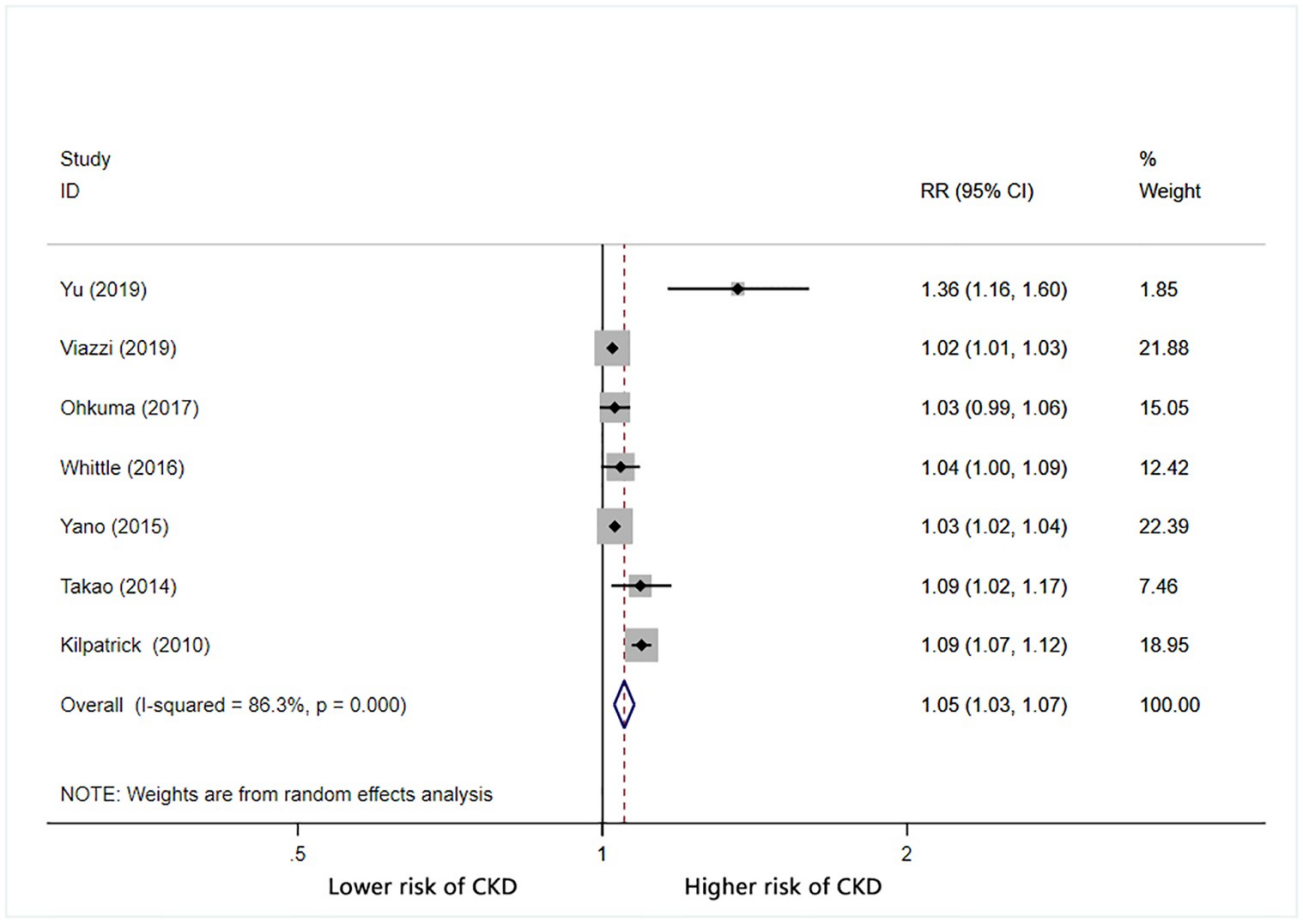
Forest plot of relationship between 1 mmHg increment in SD-SBP and risk of CKD. Boxes represent the Relative risk (RR) and lines represent the 95% Confidence Intervals (CIs) for individual studies. The area of each square is proportional to study weight. The diamonds and their width represent the pooled RRs and the 95% CIs, respectively.

**Fig 3:**
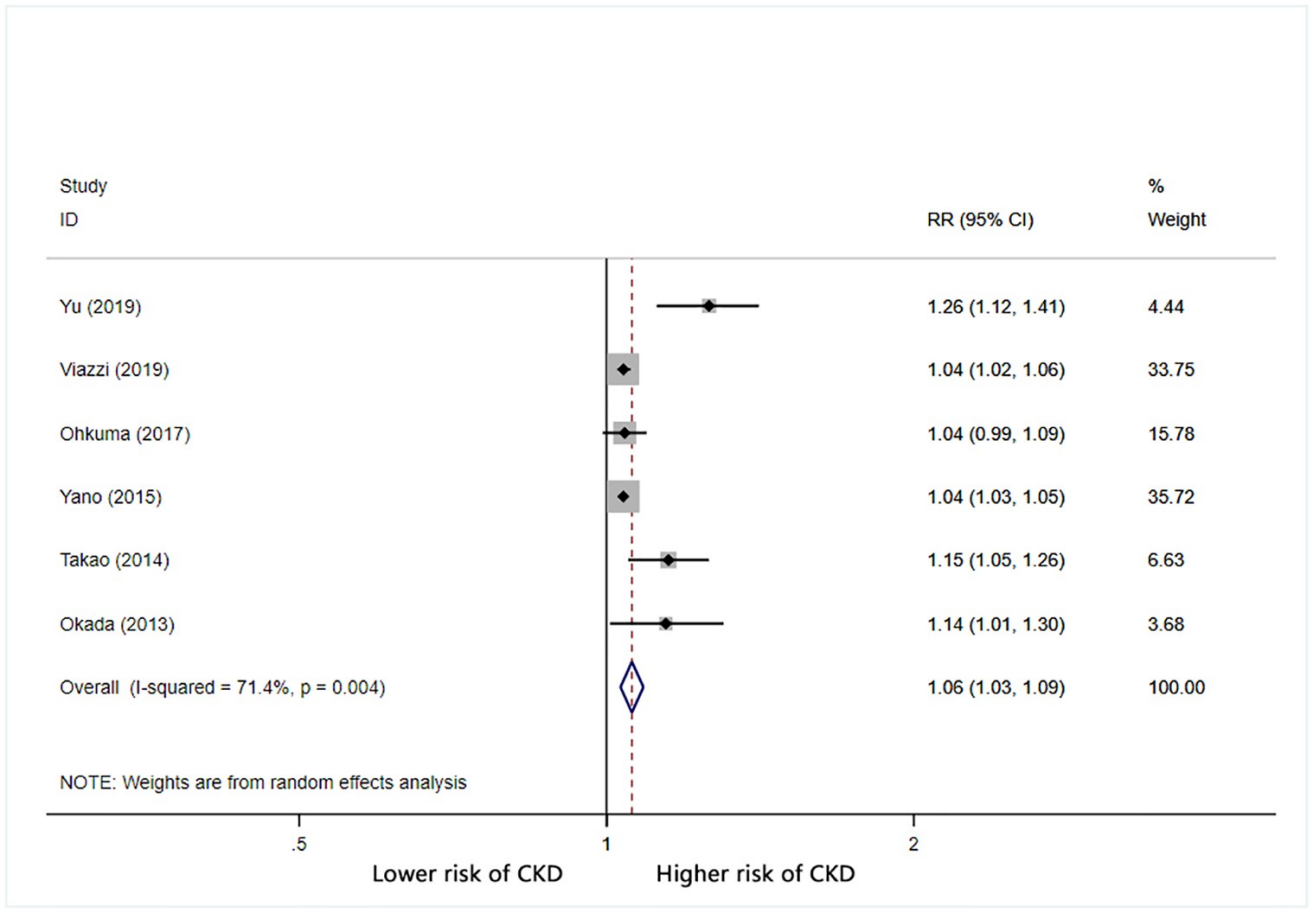
Forest plot of relationship between 1% increment in CV-SBP and risk of CKD. Boxes represent the Relative risk (RR) and lines represent the 95% Confidence Intervals (CIs) for individual studies. The area of each square is proportional to study weight. The diamonds and their width represent the pooled RRs and the 95% CIs, respectively.

**Fig 4:**
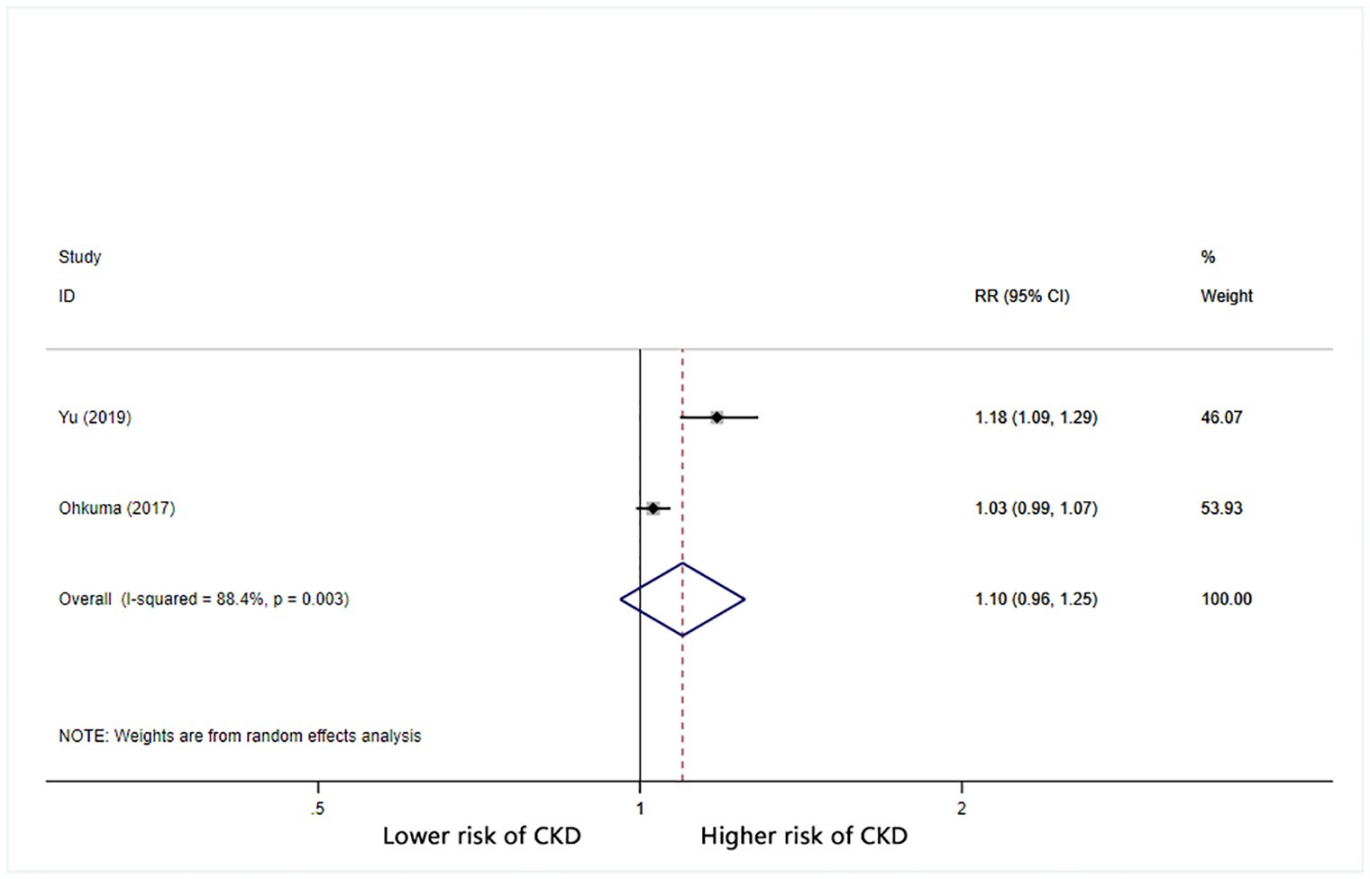
Forest plot of relationship between 1 increment in VIM-SBP and risk of CKD. Boxes represent the Relative risk (RR) and lines represent the 95% Confidence Intervals (CIs) for individual studies. The area of each square is proportional to study weight. The diamonds and their width represent the pooled RRs and the 95% CIs, respectively.

Furthermore, we calculated the combined RR (95% CI) for CKD based on increases in BPV. For each 1 mmHg increase in systolic BP SD, the combined RR for CKD was 1.05 (95% CI: 1.03–1.07) with significant heterogeneity (I^2^ = 86.3%) (Fig 2). For each 1% increase in systolic BP CV, the combined RR for CKD was 1.06 (95% CI: 1.03–1.09) with significant heterogeneity (I^2^ = 71.4%) (Fig 3). For each unit increase in systolic BP VIM, the combined RR for CKD was 1.1 (95% CI: 0.96–1.25) with significant heterogeneity (I^2^ = 88.4%) (Fig 4).

**Fig 5:**
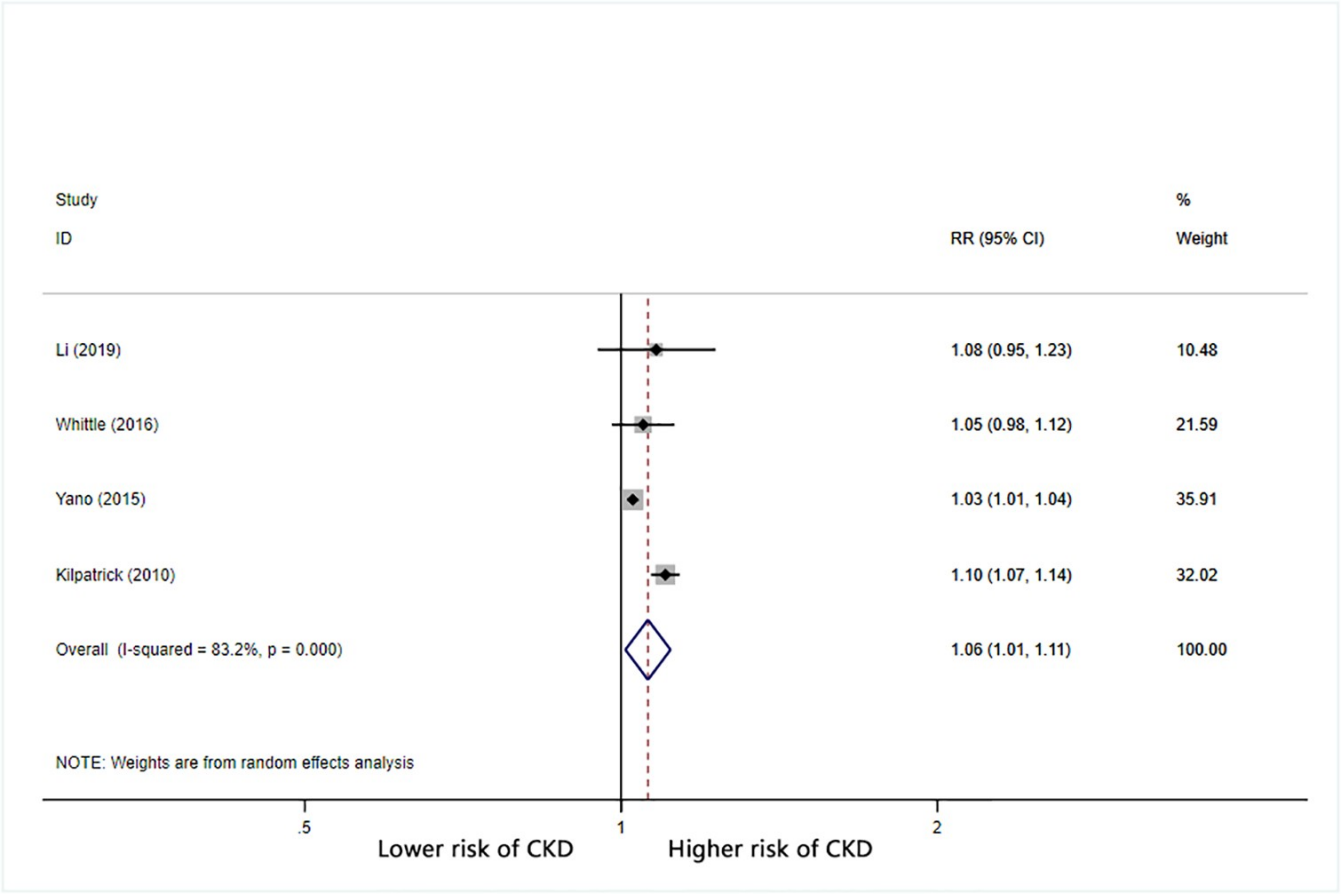
Forest plot of relationship between 1 mmHg increment in SD-DBP and risk of CKD. Boxes represent the Relative risk (RR) and lines represent the 95% Confidence Intervals (CIs) for individual studies. The area of each square is proportional to study weight. The diamonds and their width represent the pooled RRs and the 95% CIs, respectively.

**Fig 6:**
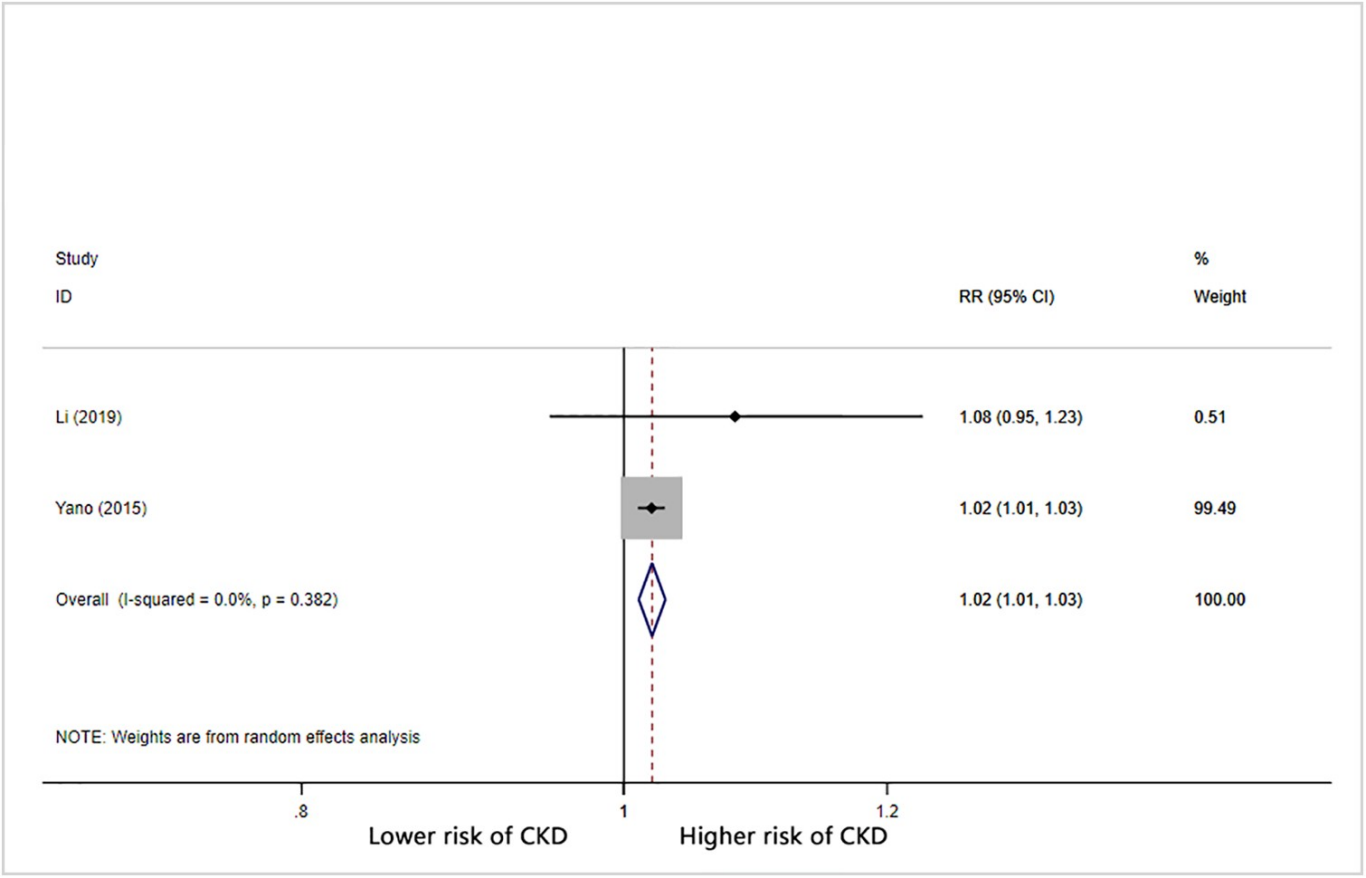
Forest plot of relationship between 1% increment in CV-DBP and risk of CKD. Boxes represent the Relative risk (RR) and lines represent the 95% Confidence Intervals (CIs) for individual studies. The area of each square is proportional to study weight. The diamonds and their width represent the pooled RRs and the 95% CIs, respectively.

Visit-to-visit diastolic BPV (DBPV) was defined using SD and CV. The risk of CKD was significantly greater in patients with high baseline DBPV than in patients with low baseline DBPV (SD [RR = 1.14, 95% CI: 1.05–1.24; I^2^ = 88.7%; S4 Fig in S1 Data] and CV [RR = 1.18, 95% CI: 0.99–1.41; I^2^ = 95.6%; S5 Fig in S1 Data]). Only one study reported DBPV using VIM; thus, we did not perform meta-analysis on the relationship of diastolic BP VIM with CKD. The combined RR (95% CI) per 1 mmHg increase in diastolic BP SD for CKD was 1.06 (95% CI: 1.01–1.11) with significant heterogeneity (I^2^ = 83.2%) (Fig 5). For each 1% increase in diastolic BP CV, the overall RR was 1.06 (95% CI: 1.03–1.09) with significant heterogeneity (I^2^ = 71.4%) (Fig 6).

### Stratifying analysis

We performed subgroup analysis of the studies based on follow-up duration, country, population, and number of subjects (Table 2). In these analyses, the association of systolic BP SD with CKD remained; however, the heterogeneity also remained. The observational design of the studies might have been the major source of heterogeneity.

**Table 2:** Subgroup analyses of 1mmHg increment in SD-SBP and risk of CKD.

	Subgroup	Studies, No.	RR (95% CI)	Heterogeneity
Follow-up duration	≤5	4	1.03(1.01, 1.05)	76.5%
	>5	3	1.07(1.02, 1.12)	77.0%
Country	Asia	3	1.11(1.00, 1.23)	86.0%
	Non-Asia	3	1.05(1.00, 1.10)	92.9%
Population	Diabetic	5	1.07(1.02, 1.12)	90.3%
	Non-diabetic	2	1.03(1.02, 1.04)	0.0%
No. of subjects	≤10000	3	1.07(1.02, 1.12)	77.0%
	>10000	4	1.03(1.01, 1.05)	76.5%

### Bias and sensitivity analysis

**Fig 7:**
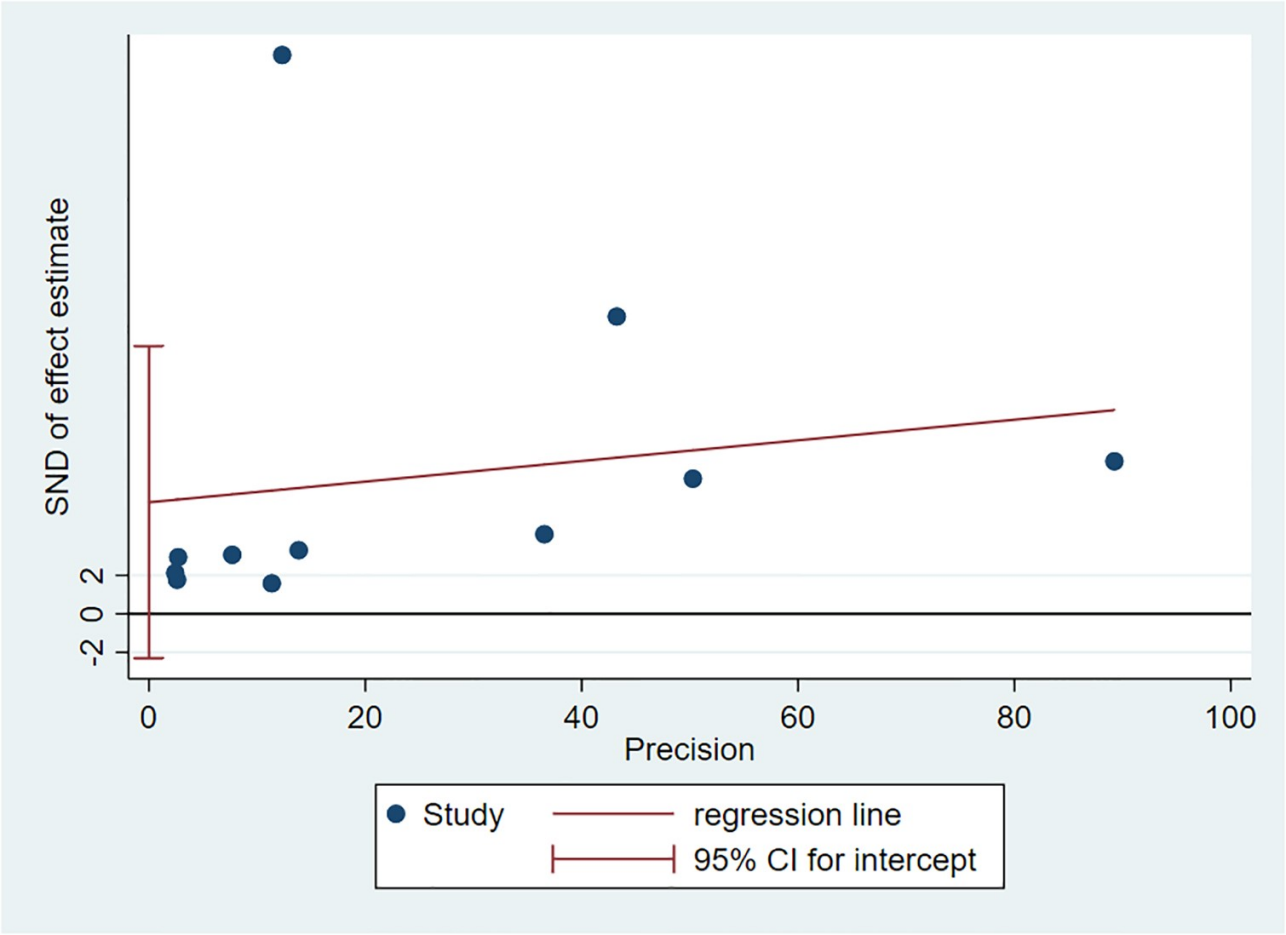
Publication bias using Egger test.

**Fig 8:**
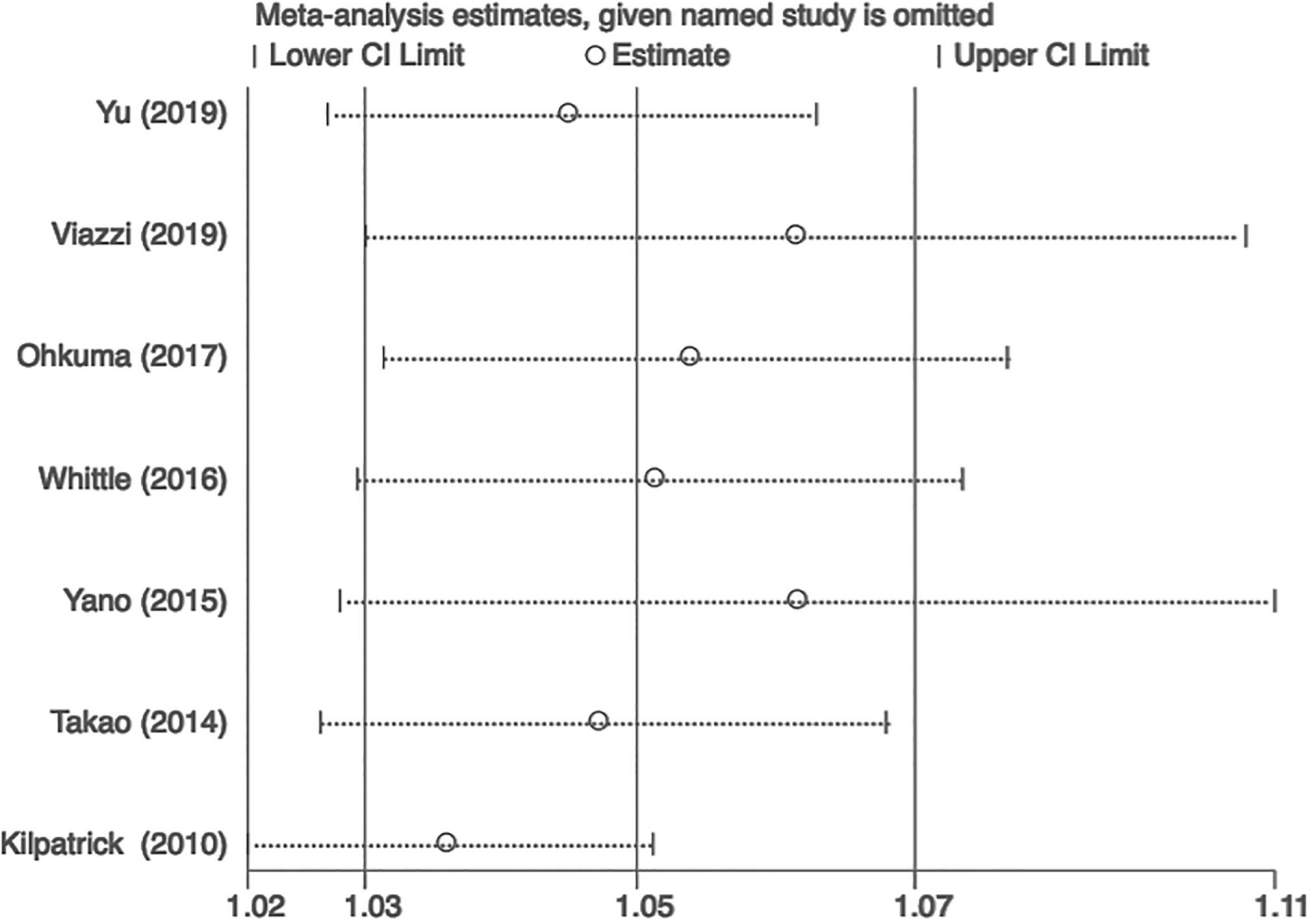
The sensitivity analysis regarding the BPV SD for CKD.

There was no evidence of publication bias regarding the BPV SD for CKD, as determined by Egger’s linear regression test (Fig 7). The results remained consistent during sensitivity analysis, indicating that the meta-analysis was stable (Fig 8).

## Discussion

To the best of our knowledge, this is the first systematic review and meta-analysis to explore the potential relationship between visit-to-visit BPV and CKD. Our study demonstrated that high visit-to-visit BPV may indicate an increased risk of CKD. Patients with higher SBPV had a 5% higher risk of CKD for each 1 mmHg increase in SD compared to patients with lower SBPV. We also used CV and VIM to examine the association between SBPV and risk of CKD; the corresponding risk ratios also showed increased risk of CKD. This association remained significant in analysis of the relationship between visit-to-visit DBPV and the risk of CKD. Our results suggest that visit-to-visit BPV is an important clinical factor for predicting CKD.

BP is a physiological parameter that reflects hemodynamic status, and it is characterized by significant changes over time. The magnitude of BP variation was initially suspected to interfere with the accuracy of BP status assessments in individual patients. However, BPV is increasingly recognized as an important pathophysiological phenomenon. Visit-to-visit BPV is presumed to reflect a variety of mechanisms, such as fluctuations in activation of the renin–angiotensin–aldosterone system, overactivity of the central sympathetic nervous system, increased secretion of vasoactive compound, and/or increased arterial stiffness, as well as environmental and psychological conditions (e.g., physical activity and psychological stress) [5]. BPV is a powerful marker of cardiovascular and renal complications regardless of average BP level [41]. In previous studies, increased visit-to-visit BPV, independent of the mean BP, was linked to increases in all-cause mortality, as well as fatal and non-fatal cardiovascular disease events in the general population [42–44]. A significant relationship has been reported between the progression of renal insufficiency and higher visit-to-visit BPV in patients with CKD [45, 46].

Even mild renal impairment constitutes a prominent risk factor for cardiovascular disease, infection, cognitive impairment, and reduced physical function [47]. Our findings suggest that visit-to-visit BPV is an independent predictor of early renal impairment. Potential biological mechanisms underlying the relationship between visit-to-visit BPV and CKD may include fluctuations in renal blood flow, changes in aortic hypertrophy and remodeling, onset of endothelial dysfunction, activation of the renin–angiotensin system, activation of inflammatory cytokines, changes in oxidative stress or extracellular matrix deposition, and onset of glomerular sclerosis [48–52].

Several limitations might have contributed to the significant heterogeneity detected in this meta-analysis. Differences in population, sample size, follow-up duration, incomplete matching, country of origin, or methodology may have caused heterogeneity; of these factors, the observational study design of the included studies might have been the most prominent source. Methodological factors including number of visits, time interval between visits, and assessments by different doctors may have also contributed to the heterogeneity.

Taken together, the findings in this systematic review and meta-analysis suggest that increased visit-to-visit SBPV is an independent risk factor for CKD. However, significant heterogeneity was present; thus, future prospective studies are needed to confirm our findings. Our current findings indicate that treatment of hypertension should control BP levels and prevent abnormal fluctuations in BP to restore normal BP rhythm. Additional clinical studies are needed to evaluate the normal reference values of visit-to-visit BPV for clinical practice and identify more effective treatment approaches for reduction of BPV.

## Supporting information

S1 Data (DOCX)

S1 Checklist PRISMA 2009 checklist. (DOC)

## References

[1] Saran R, Robinson B, Abbott KC, Agodoa LYC, Bragg-Gresham J, Balkrishnan R, et al US Renal Data System 2018 Annual Data Report: Epidemiology of Kidney Disease in the United States. American journal of kidney diseases: the official journal of the National Kidney Foundation. 2019;73(3s1):A7–a8. 10.1053/j.ajkd.2019.01.001 . 30798791 PMC6620109

[2] Zhang L, Wang F, Wang L, Wang W, Liu B, Liu J, et al Prevalence of chronic kidney disease in China: a cross-sectional survey. The Lancet. 2012;379(9818):815–22. 10.1016/S0140-6736(12)60033-6 22386035

[3] Kannel WB. Blood pressure as a cardiovascular risk factor: prevention and treatment. JAMA: the journal of the American Medical Association. 275(20):1571–6. . 8622248

[4] Stevens PE, Levin A. Evaluation and management of chronic kidney disease: synopsis of the kidney disease: improving global outcomes 2012 clinical practice guideline. Annals of internal medicine. 2013;158(11):825–30. 23732715 10.7326/0003-4819-158-11-201306040-00007

[5] Parati G, Ochoa JE, Lombardi C, Bilo G. Assessment and management of blood-pressure variability. Nature reviews Cardiology. 2013;10(3):143–55. 10.1038/nrcardio.2013.1 . 23399972

[6] Wang H, Li M, Xie SH, Oyang YT, Yin M, Bao B, et al Visit-to-visit Systolic Blood Pressure Variability and Stroke Risk: A Systematic Review and Meta-analysis. Current medical science. 2019;39(5):741–7. 10.1007/s11596-019-2100-9 . 31612391

[7] Chiriacò M, Pateras K, Virdis A, Charakida M, Kyriakopoulou D, Nannipieri M, et al Association between blood pressure variability, cardiovascular disease and mortality in type 2 diabetes: A systematic review and meta-analysis. Diabetes, obesity & metabolism. 2019 10.1111/dom.13828 . 31282073

[8] Wang J, Shi X, Ma C, Zheng H, Xiao J, Bian H, et al Visit-to-visit blood pressure variability is a risk factor for all-cause mortality and cardiovascular disease: a systematic review and meta-analysis. Journal of hypertension. 2017;35(1):10–7. Epub 2016/12/03. 10.1097/hjh.0000000000001159 . 27906836

[9] Shafi T, Sozio SM, Bandeen-Roche KJ, Ephraim PL, Luly JR, Peter WLS, et al Predialysis systolic BP variability and outcomes in hemodialysis patients. Journal of the American Society of Nephrology. 2014;25(4):799–809. 24385593 10.1681/ASN.2013060667 PMC3968504

[10] Okada H, Fukui M, Tanaka M, Matsumoto S, Mineoka Y, Nakanishi N, et al Visit-to-visit blood pressure variability is a novel risk factor for the development and progression of diabetic nephropathy in patients with type 2 diabetes. Diabetes care. 2013;36(7):1908–12. Epub 2013/01/24. 10.2337/dc12-2087 23340892 PMC3687293

[11] Noshad S, Mousavizadeh M, Mozafari M, Nakhjavani M, Esteghamati A. Visit-to-visit blood pressure variability is related to albuminuria variability and progression in patients with type 2 diabetes. Journal of human hypertension. 2014;28(1):37–43. Epub 2013/07/19. 10.1038/jhh.2013.36 . 23863801

[12] Hata J, Arima H, Rothwell PM, Woodward M, Zoungas S, Anderson C, et al Effects of visit-to-visit variability in systolic blood pressure on macrovascular and microvascular complications in patients with type 2 diabetes mellitus: the ADVANCE trial. Circulation. 2013;128(12):1325–34. Epub 2013/08/09. 10.1161/circulationaha.113.002717 . 23926207

[13] Kilpatrick ES, Rigby AS, Atkin SL. The role of blood pressure variability in the development of nephropathy in type 1 diabetes. Diabetes care. 2010;33(11):2442–7. 10.2337/dc10-1000 . 20798339 PMC2963509

[14] Ceriello A, De Cosmo S, Rossi MC, Lucisano G, Genovese S, Pontremoli R, et al Variability in HbA1c, blood pressure, lipid parameters and serum uric acid, and risk of development of chronic kidney disease in type 2 diabetes. Diabetes, obesity & metabolism. 2017;19(11):1570–8. Epub 2017/04/23. 10.1111/dom.12976 . 28432733

[15] Yokoyama H, Kanno S, Takahashi S, Yamada D, Itoh H, Saito K, et al Determinants of decline in glomerular filtration rate in nonproteinuric subjects with or without diabetes and hypertension. Clin J Am Soc Nephrol. 2009;4(9):1432–40. Epub 2009/08/29. 10.2215/cjn.06511208 19713288 PMC2736691

[16] Stroup DF, Berlin JA, Morton SC, Olkin I, Williamson GD, Rennie D, et al Meta-analysis of observational studies in epidemiology: a proposal for reporting. Meta-analysis Of Observational Studies in Epidemiology (MOOSE) group. JAMA: the journal of the American Medical Association. 2000;283(15):2008–12. Epub 2000/05/02. 10.1001/jama.283.15.2008 . 10789670

[17] Chandler J, Cumpston M, Li T, Page MJ, Welch VA. Cochrane handbook for systematic reviews of interventions: John Wiley & Sons; 2019. 10.1002/14651858.ED000142 PMC10284251 31643080

[18] Moher D, Liberati A, Tetzlaff J, Altman DG. Preferred reporting items for systematic reviews and meta-analyses: the PRISMA statement. Journal of clinical epidemiology. 2009;62(10):1006–12. Epub 2009/07/28. 10.1016/j.jclinepi.2009.06.005 . 19631508

[19] Riley RD, Moons KG, Snell KI, Ensor J, Hooft L, Altman DG, et al A guide to systematic review and meta-analysis of prognostic factor studies. Bmj. 2019;364:k4597. 30700442 10.1136/bmj.k4597

[20] Hayden JA, van der Windt DA, Cartwright JL, Côté P, Bombardier C. Assessing bias in studies of prognostic factors. Annals of internal medicine. 2013;158(4):280–6. 23420236 10.7326/0003-4819-158-4-201302190-00009

[21] Symons MJ, Moore DT. Hazard rate ratio and prospective epidemiological studies. Journal of clinical epidemiology. 2002;55(9):893–9. 10.1016/s0895-4356(02)00443-2 . 12393077

[22] Loef M, Walach H. The combined effects of healthy lifestyle behaviors on all cause mortality: a systematic review and meta-analysis. Prev Med. 2012;55(3):163–70. 10.1016/j.ypmed.2012.06.017 . 22735042

[23] Berlin JA, Longnecker MP, Greenland S. Meta-analysis of epidemiologic dose-response data. Epidemiology. 1993;4(3):218–28. 10.1097/00001648-199305000-00005 . 8512986

[24] Orsini N, Li R, Wolk A, Khudyakov P, Spiegelman D. Meta-analysis for linear and nonlinear dose-response relations: examples, an evaluation of approximations, and software. Am J Epidemiol. 2012;175(1):66–73. 10.1093/aje/kwr265 . 22135359 PMC3244608

[25] Greenland S, Longnecker MP. Methods for trend estimation from summarized dose-response data, with applications to meta-analysis. Am J Epidemiol. 1992;135(11):1301–9. 10.1093/oxfordjournals.aje.a116237 . 1626547

[26] Tai C, Sun Y, Dai N, Xu D, Chen W, Wang J, et al Prognostic significance of visit-to-visit systolic blood pressure variability: a meta-analysis of 77,299 patients. Journal of clinical hypertension (Greenwich, Conn). 2015;17(2):107–15. 10.1111/jch.12484 . 25644682 PMC8031983

[27] Higgins JP, Thompson SG, Deeks JJ, Altman DG. Measuring inconsistency in meta-analyses. Bmj. 2003;327(7414):557–60. Epub 2003/09/06. 10.1136/bmj.327.7414.557 12958120 PMC192859

[28] Begg CB, Mazumdar M. Operating characteristics of a rank correlation test for publication bias. Biometrics. 1994;50(4):1088–101. Epub 1994/12/01. . 7786990

[29] Kim MK, Han K, Kim HS, Park YM, Kwon HS, Yoon KH, et al Effects of Variability in Blood Pressure, Glucose, and Cholesterol Concentrations, and Body Mass Index on End-Stage Renal Disease in the General Population of Korea. Journal of clinical medicine. 2019;8(5). Epub 2019/05/30. 10.3390/jcm8050755 31137866 PMC6571839

[30] Takao T, Suka M, Yanagisawa H, Matsuyama Y, Iwamoto Y. Predictive ability of visit-to-visit variability in HbA1c and systolic blood pressure for the development of microalbuminuria and retinopathy in people with type 2 diabetes. Diabetes Res Clin Pract. 2017;128:15–23. 10.1016/j.diabres.2017.03.027 . 28432895

[31] Yu ZB, Wang JB, Li D, Chen XY, Lin HB, Chen K. Prognostic value of visit-to-visit systolic blood pressure variability related to diabetic kidney disease among patients with type 2 diabetes. Journal of hypertension. 2019;37(7):1411–8. Epub 2019/01/15. 10.1097/hjh.0000000000002038 . 30640884

[32] Viazzi F, Bonino B, Mirijello A, Fioretto P, Giorda C, Ceriello A, et al Long-term blood pressure variability and development of chronic kidney disease in type 2 diabetes. Journal of hypertension. 2019 Epub 2019/01/10. 10.1097/hjh.0000000000001950 . 30817462

[33] Li Y, Li D, Song Y, Gao L, Fan F, Wang B, et al Visit-to-visit variability in blood pressure and the development of chronic kidney disease in treated general hypertensive patients. Nephrol Dial Transplant. 2019 Epub 2019/05/19. 10.1093/ndt/gfz093 . 31102525

[34] Bae EH, Lim SY, Han KD, Oh TR, Choi HS, Kim CS, et al Association Between Systolic and Diastolic Blood Pressure Variability and the Risk of End-Stage Renal Disease. Hypertension. 2019:Hypertensionaha11913422. Epub 2019/08/20. 10.1161/hypertensionaha.119.13422 . 31422691 PMC6756299

[35] Sohn MW, Epstein N, Huang ES, Huo Z, Emanuele N, Stukenborg G, et al Visit-to-visit systolic blood pressure variability and microvascular complications among patients with diabetes. Journal of diabetes and its complications. 2017;31(1):195–201. Epub 2016/09/28. 10.1016/j.jdiacomp.2016.09.003 27671535 PMC5209256

[36] Ohkuma T, Woodward M, Jun M, Muntner P, Hata J, Colagiuri S, et al Prognostic Value of Variability in Systolic Blood Pressure Related to Vascular Events and Premature Death in Type 2 Diabetes Mellitus: The ADVANCE-ON Study. Hypertension. 2017;70(2):461–8. Epub 2017/06/07. 10.1161/hypertensionaha.117.09359 . 28584014

[37] Whittle J, Lynch AI, Tanner RM, Simpson LM, Davis BR, Rahman M, et al Visit-to-Visit Variability of BP and CKD Outcomes: Results from the ALLHAT. Clin J Am Soc Nephrol. 2016;11(3):471–80. Epub 2016/02/26. 10.2215/cjn.04660415 26912544 PMC4791814

[38] Gosmanova EO, Mikkelsen MK, Molnar MZ, Lu JL, Yessayan LT, Kalantar-Zadeh K, et al Association of Systolic Blood Pressure Variability With Mortality, Coronary Heart Disease, Stroke, and Renal Disease. Journal of the American College of Cardiology. 2016;68(13):1375–86. Epub 2016/09/24. 10.1016/j.jacc.2016.06.054 27659458 PMC5117818

[39] Yano Y, Fujimoto S, Kramer H, Sato Y, Konta T, Iseki K, et al Long-Term Blood Pressure Variability, New-Onset Diabetes Mellitus, and New-Onset Chronic Kidney Disease in the Japanese General Population. Hypertension. 2015;66(1):30–6. Epub 2015/05/20. 10.1161/hypertensionaha.115.05472 . 25987664

[40] Takao T, Matsuyama Y, Yanagisawa H, Kikuchi M, Kawazu S. Visit-to-visit variability in systolic blood pressure predicts development and progression of diabetic nephropathy, but not retinopathy, in patients with type 2 diabetes. Journal of diabetes and its complications. 2014;28(2):185–90. Epub 2013/12/18. 10.1016/j.jdiacomp.2013.11.003 . 24332763

[41] Muntner P, Joyce C, Levitan EB, Holt E, Shimbo D, Webber LS, et al Reproducibility of visit-to-visit variability of blood pressure measured as part of routine clinical care. Journal of hypertension. 2011;29(12):2332–8. Epub 2011/10/26. 10.1097/HJH.0b013e32834cf213 22025235 PMC4867495

[42] Muntner P, Shimbo D, Tonelli M, Reynolds K, Arnett DK, Oparil S. The relationship between visit-to-visit variability in systolic blood pressure and all-cause mortality in the general population: findings from NHANES III, 1988 to 1994. Hypertension. 2011;57(2):160–6. 10.1161/hypertensionaha.110.162255 . 21200000

[43] Stevens SL, Wood S, Koshiaris C, Law K, Glasziou P, Stevens RJ, et al Blood pressure variability and cardiovascular disease: systematic review and meta-analysis. Bmj. 2016;354:i4098 10.1136/bmj.i4098 . 27511067 PMC4979357

[44] Mehlum MH, Liestøl K, Kjeldsen SE, Julius S, Hua TA, Rothwell PM, et al Blood pressure variability and risk of cardiovascular events and death in patients with hypertension and different baseline risks. Eur Heart J. 2018;39(24):2243–51. 10.1093/eurheartj/ehx760 . 29365085

[45] Chang TI, Tabada GH, Yang J, Tan TC, Go AS. Visit-to-visit variability of blood pressure and death, end-stage renal disease, and cardiovascular events in patients with chronic kidney disease. J Hypertens. 2016;34(2):244–52. 10.1097/hjh.0000000000000779 . 26599220 PMC4818097

[46] Yokota K, Fukuda M, Matsui Y, Hoshide S, Shimada K, Kario K. Impact of visit-to-visit variability of blood pressure on deterioration of renal function in patients with non-diabetic chronic kidney disease. Hypertens Res. 2013;36(2):151–7. 10.1038/hr.2012.145 . 23013884

[47] Levey AS, Coresh J. Chronic kidney disease. Lancet. 2012;379(9811):165–80. 10.1016/s0140-6736(11)60178-5 . 21840587

[48] Sun Y, Fan J, Chai D, Zhang M. Oxidative Stress Is Involved in the Renal Dysfunction Induced by Sinoaortic Denervation in Rats. Chemical & pharmaceutical bulletin. 2016;64(10):1458–65. 10.1248/cpb.c16-00318 . 27489120

[49] Wu F, Feng JZ, Qiu YH, Yu FB, Zhang JZ, Zhou W, et al Activation of receptor for advanced glycation end products contributes to aortic remodeling and endothelial dysfunction in sinoaortic denervated rats. Atherosclerosis. 2013;229(2):287–94. 10.1016/j.atherosclerosis.2013.04.033 . 23880178

[50] DiBona GF, Jones SY. Dynamic analysis of renal nerve activity responses to baroreceptor denervation in hypertensive rats. Hypertension. 2001;37(4):1153–63. 10.1161/01.hyp.37.4.1153 . 11304518

[51] Aoki Y, Kai H, Kajimoto H, Kudo H, Takayama N, Yasuoka S, et al Large blood pressure variability aggravates arteriolosclerosis and cortical sclerotic changes in the kidney in hypertensive rats. Circ J. 2014;78(9):2284–91. 10.1253/circj.cj-14-0027 . 24976508

[52] Freitas FF, Araujo G, Porto ML, Freitas FP, Graceli JB, Balarini CM, et al Increased Blood Pressure Variability Prior to Chronic Kidney Disease Exacerbates Renal Dysfunction in Rats. Frontiers in physiology. 2016;7:428 10.3389/fphys.2016.00428 . 27721797 PMC5034010

