# Supplementary material for: Visit-to-visit blood pressure variability and risk of chronic kidney disease: A systematic review and meta-analyses
Source: PLoS One. 2020 May 29;15(5):e0233233. doi: 10.1371/journal.pone.0233233 (PMC7259502; doi:10.1371/journal.pone.0233233)
Supplement: S1 Data — (DOCX) [file pone.0233233.s001.docx]

**Search strategy:**

**Medline via Ovid:**

1. *Blood Pressure/

2. (blood pressure or bp or sbp or dbp).ti,ab.

3. (variability or variabilities).ti,ab.

4. variation?.ti,ab.

5. ((between or within) adj3 visit?).ti,ab.

6. "visit to visit".ti,ab.

7. ((between or within) adj day?).ti,ab.

8. ("day to day" or "day by day").ti,ab.

9. "measure* to measure*".ti,ab.

10. "reading? to reading?".ti,ab.

11. repeat* measure*.ti,ab.

12. ((daytime or day-time or diurnal) adj5 (night-time or nocturnal)).ti,ab.

13. (((daytime or day-time or diurnal) adj5 (blood pressure or bp or sbp or sbp)) and ((night-time or nocturnal) adj5 (blood pressure or bp or sbp or sbp))).ti,ab.

14. (dipping or dipper? or nondipping or nondipper? or non-dipping or non-dipper?).ti,ab.

15. within subject?.ti,ab.

16. ((blood pressure or bp or sbp or dbp) adj5 (variabilit* or variation?)).ti,ab.

17. chronic kidney disease.mp.

18. ((chronic or diabetic or advanced) adj kidney).tw.

19. ((kidney or renal) adj (disease or insufficiency)).ti,ab.

20. ckd.mp.

21. ((chronic or progressive) adj (kidney or renal or ur?emi$)).ti,ab.

22. *diabetic nephropathy/

23. ((end stage or endstage) adj (kidney or renal)).ti,ab.

24. *chronic kidney failure/

25. (macroalbuminuria or microalbuminuria or albuminuria).tw.

26. ((kidney or renal) and (ckf or crd or crf or eskd or eskf or esrd or esrf)).mp.

27. ((exp animal/ or exp vertebrate/ or exp invertebrate/) not human/) or animal experiment/ or animal model/ or animal tissue/ or animal cell/ or nonhuman/

28. exp risk/

29. prevalence/

30. incidence/

31. (risk* or prevalence* or incidence* or predict* or associat*).ti,ab.

32. 1 or 2

33. or/3-15

34. 32 and 33

35. 16 or 34

36. or/17-26

37. or/28-31

38. 35 and 36 and 37

39. 38 not 27

**Embase via Ovid:**

1. *Blood Pressure/

2. exp *blood pressure measurement/

3. (blood pressure or bp or sbp or dbp).ti,ab.

4. (variability or variabilities).ti,ab.

5. variation?.ti,ab.

6. ((between or within) adj3 visit?).ti,ab.

7. "visit to visit".ti,ab.

8. ((between or within) adj day?).ti,ab.

9. ("day to day" or "day by day").ti,ab.

10. "measure* to measure*".ti,ab.

11. "reading? to reading?".ti,ab.

12. repeat* measure*.ti,ab.

13. ((daytime or day-time or diurnal) adj5 (night-time or nocturnal)).ti,ab.

14. (((daytime or day-time or diurnal) adj5 (blood pressure or bp or sbp or sbp)) and ((night-time or nocturnal) adj5 (blood pressure or bp or sbp or sbp))).ti,ab.

15. (dipping or dipper? or nondipping or nondipper? or non-dipping or non-dipper?).ti,ab.

16. within subject?.ti,ab.

17. blood pressure variability/

18. ((blood pressure or bp or sbp or dbp) adj5 (variabilit* or variation?)).ti,ab.

19. chronic kidney disease.mp.

20. ((chronic or diabetic or advanced) adj kidney).tw.

21. ((kidney or renal) adj (disease or insufficiency)).ti,ab.

22. ckd.mp.

23. ((chronic or progressive) adj (kidney or renal or ur?emi$)).ti,ab.

24. *diabetic nephropathy/

25. ((end stage or endstage) adj (kidney or renal)).ti,ab.

26. *chronic kidney failure/

27. (macroalbuminuria or microalbuminuria or albuminuria).tw.

28. ((kidney or renal) and (ckf or crd or crf or eskd or eskf or esrd or esrf)).mp.

29. ((exp animal/ or exp vertebrate/ or exp invertebrate/) not human/) or animal experiment/ or animal model/ or animal tissue/ or animal cell/ or nonhuman/

30. exp *risk/

31. *prevalence/

32. *incidence/

33. (risk* or prevalence* or incidence* or predict* or associat*).ti,ab.

34. or/1-3

35. or/4-16

36. 34 and 35

37. 17 or 18 or 36

38. or/19-28

39. or/30-33

40. 37 and 38 and 39

41. 40 not 29

**The Cochrane Library:**

1. blood pressure

2. bp or sbp or dbp

3. variability or variabilities or variation

4. renal or kidney

5. nephropathy

6. macroalbuminuria

7. microalbuminuria

8. albuminuria

9. uremia

10. ckd or esrd or crf

11. #1 or #2

12. #4 or #5 or #6 or #7 or #8 or #9 or #10

13. #11 and #3 and #12

**S1 table. Risk of bias assessment for cohort studies conducted with the Quality in Prognostic Studies (QUIPS) tool.**

| **Study** | **Study participation** | **Study attrition** | **Prognostic factor measurement** | **Outcome measurement** | **Study confounding** | **Statistical analysis and reporting** |
| --- | --- | --- | --- | --- | --- | --- |
| **Yu 2019** | **L** | **H** | **L** | **L** | **L** | **L** |
| **Viazzi 2019** | **L** | **H** | **L** | **L** | **L** | **L** |
| **Li 2019** | **L** | **M** | **L** | **L** | **L** | **L** |
| **Bae 2019** | **M** | **H** | **M** | **L** | **L** | **L** |
| **Sohn 2016** | **M** | **H** | **L** | **L** | **L** | **L** |
| **Ohkuma 2017** | **L** | **L** | **L** | **L** | **L** | **L** |
| **Ceriello 2017** | **M** | **H** | **M** | **L** | **M** | **L** |
| **Whittle 2016** | **L** | **H** | **L** | **L** | **L** | **L** |
| **Gosmanova 2016** | **M** | **H** | **L** | **M** | **L** | **L** |
| **Yano 2015** | **L** | **H** | **L** | **L** | **L** | **L** |
| **Takao 2014** | **M** | **H** | **L** | **L** | **M** | **L** |
| **Noshad 2014** | **M** | **L** | **H** | **H** | **H** | **M** |
| **Okada 2013** | **M** | **H** | **L** | **M** | **M** | **M** |
| **Kilpatrick 2010** | **L** | **H** | **L** | **L** | **H** | **L** |

**
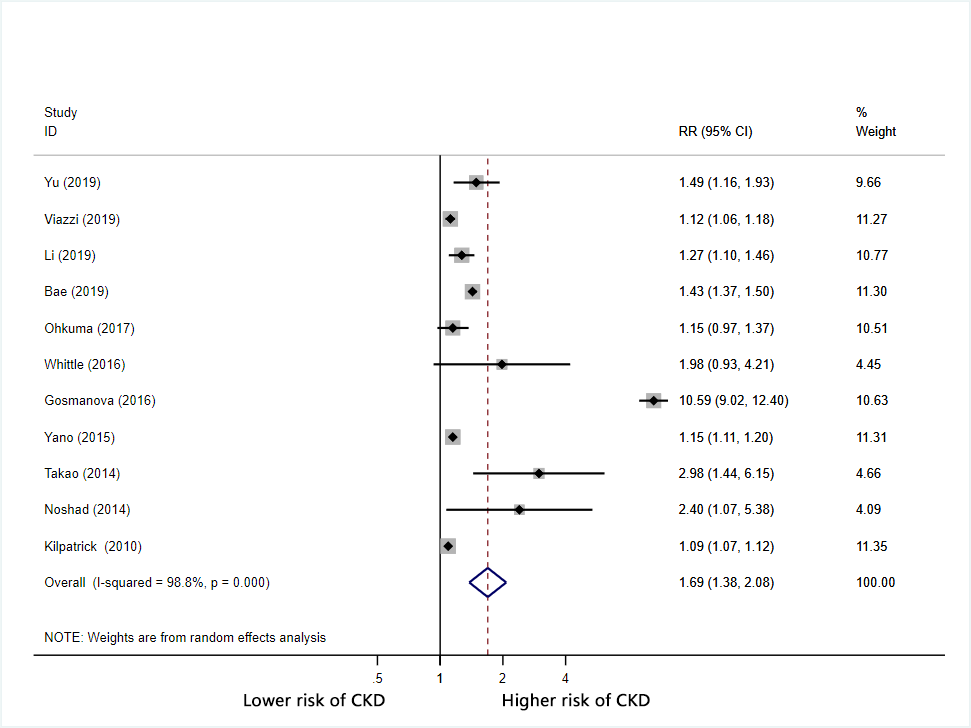
**

**S1 Fig. Forest plot comparing SD-SBP and risk of CKD.** Boxes represent the Relative risk (RR) and lines represent the 95% Confidence Intervals (CIs) for individual studies. The diamonds and their width represent the pooled RRs and the 95% CIs, respectively.


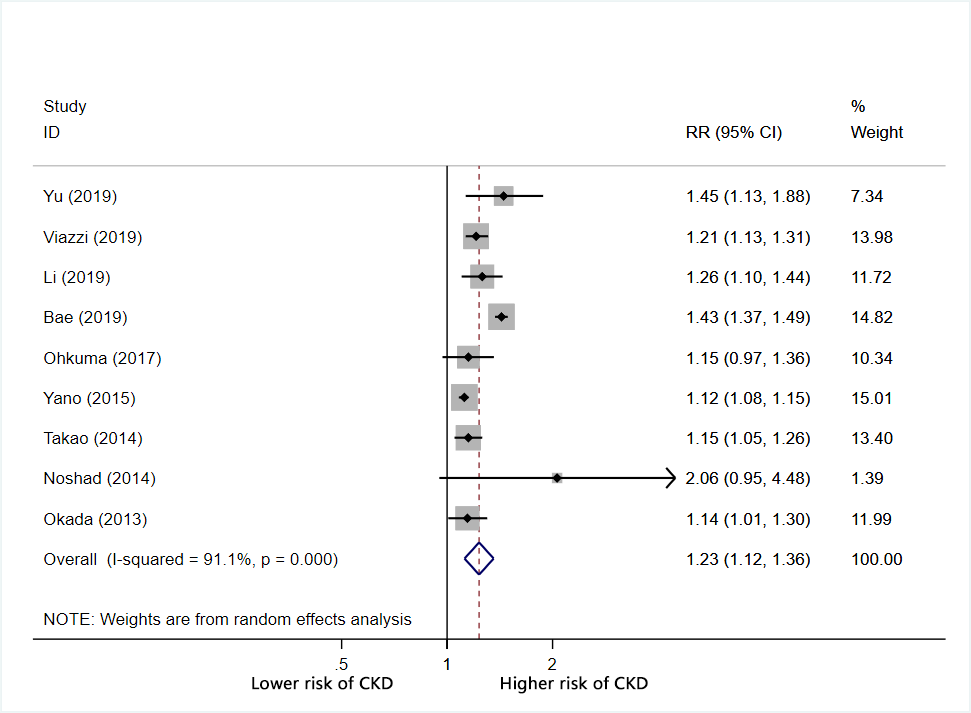


**S2 Fig. Forest plot comparing CV-SBP and risk of CKD**. Boxes represent the Relative risk (RR) and lines represent the 95% Confidence Intervals (CIs) for individual studies. The diamonds and their width represent the pooled RRs and the 95% CIs, respectively.


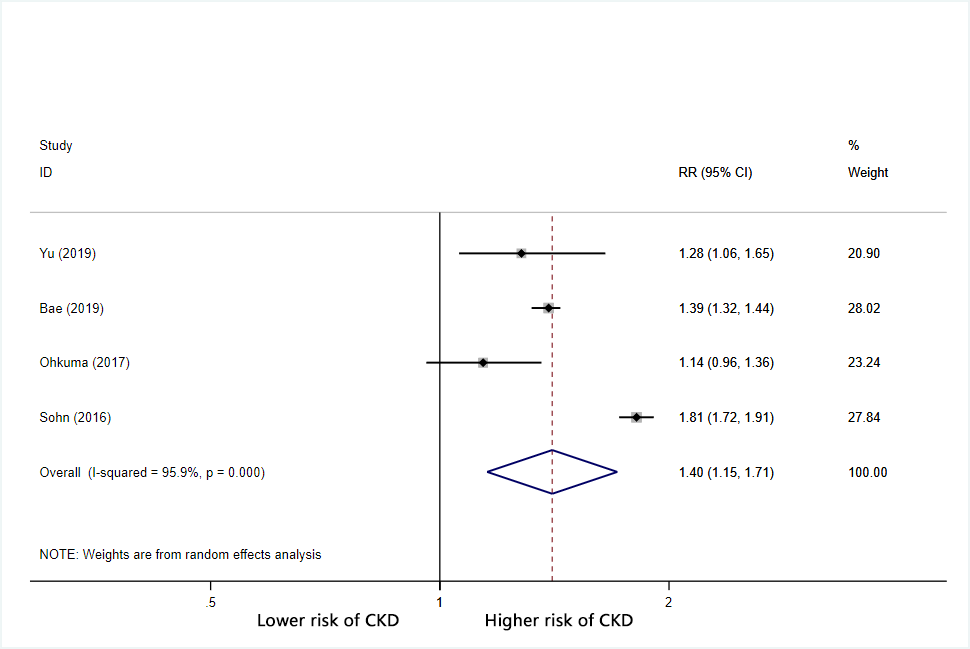


**S3 Fig. Forest plot comparing VIM-SBP and risk of CKD.** Boxes represent the Relative risk (RR) and lines represent the 95% Confidence Intervals (CIs) for individual studies. The diamonds and their width represent the pooled RRs and the 95% CIs, respectively.


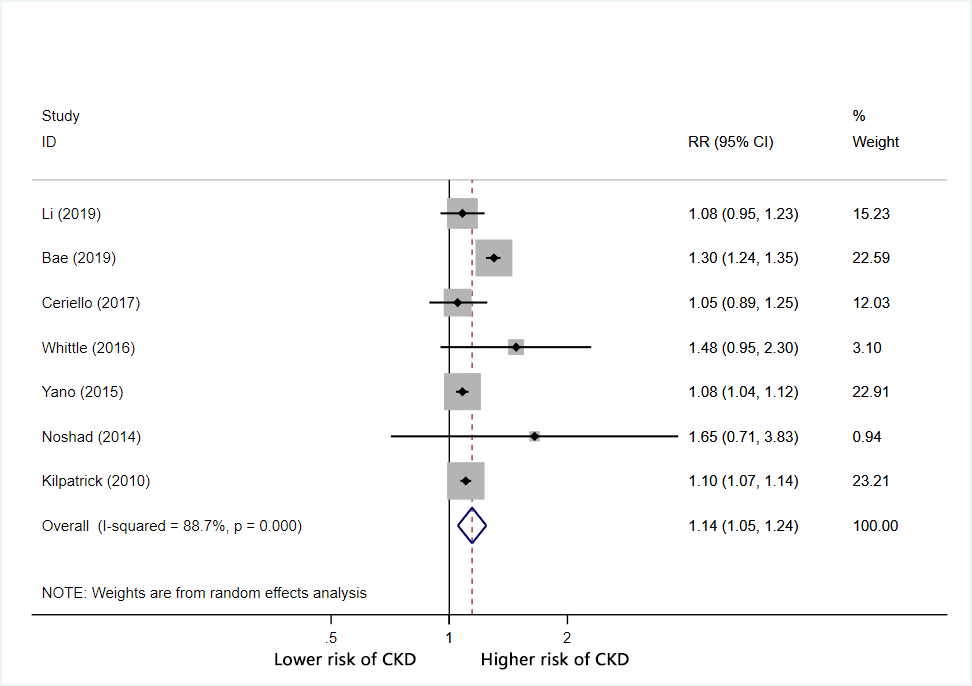


**S4 Fig. Forest plot comparing SD-DBP and risk of CKD.** Boxes represent the Relative risk (RR) and lines represent the 95% Confidence Intervals (CIs) for individual studies. The diamonds and their width represent the pooled RRs and the 95% CIs, respectively.


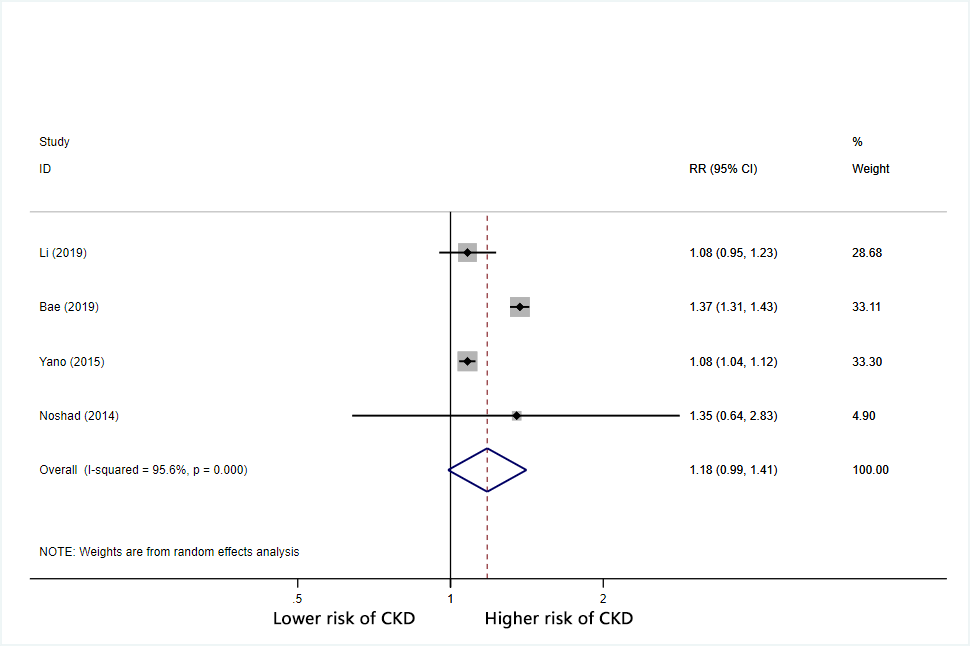


**S5 Fig. Forest plot comparing CV-DBP and risk of CKD.** Boxes represent the Relative risk (RR) and lines represent the 95% Confidence Intervals (CIs) for individual studies. The diamonds and their width represent the pooled RRs and the 95% CIs, respectively.
